# Population-Based Analysis of Invasive Fungal Infections, France, 2001–2010

**DOI:** 10.3201/eid2007.140087

**Published:** 2014-07

**Authors:** Dounia Bitar, Olivier Lortholary, Yann Le Strat, Javier Nicolau, Bruno Coignard, Pierre Tattevin, Didier Che, Françoise Dromer

**Affiliations:** Insitut de Veille Sanitaire, Saint Maurice, France (D. Bitar, Y. Le Strat, J. Nicolau, B. Coignard, D. Che);; Institut Pasteur, Paris, France (O. Lortholary, F. Dromer);; Centre National de la Recherche Scientifique, Paris (O. Lortholary, F. Dromer);; Université Paris Descartes, Paris (O. Lortholary);; CHUPontchaillou, Rennes, France (P. Tattevin)

**Keywords:** Invasive mycosis, candidemia, *Pneumocystis jirovecii*, aspergillosis, cryptococcosis, mucormycosis

## Abstract

To determine the epidemiology and trends of invasive fungal infections (IFIs) in France, we analyzed incidence, risk factors, and in-hospital death rates related to the most frequent IFIs registered in the national hospital discharge database during 2001–2010. The identified 35,876 IFI cases included candidemia (43.4%), *Pneumocystis jirovecii* pneumonia (26.1%), invasive aspergillosis (IA, 23.9%), cryptococcosis (5.2%), and mucormycosis (1.5%). The overall incidence was 5.9/100,000 cases/year and the mortality rate was 27.6%; both increased over the period (+1.5%, +2.9%/year, respectively). Incidences substantially increased for candidemia, IA, and mucormycosis. *Pneumocystis jirovecii* pneumonia incidence decreased among AIDS patients (−14.3%/year) but increased in non-HIV–infected patients (+13.3%/year). Candidemia and IA incidence was increased among patients with hematologic malignancies (>+4%/year) and those with chronic renal failure (>+10%/year). In-hospital deaths substantially increased in some groups, e.g., in those with hematologic malignancies. IFIs occur among a broad spectrum of non–HIV-infected patients and should be a major public health priority.

## Abstract 2

These infections are underrecognized as a cause of death in the general population and high-risk groups.

Invasive fungal infections (IFI) are reportedly increasing in many countries, especially candidemia and invasive aspergillosis (IA) among immunocompromised patients (*1*–*4*). Conversely, a decline of AIDS-associated *Pneumocystis jirovecii* pneumonia (Pjp) and cryptococcosis has been observed in Western countries since the advent of highly active antiretroviral treatments (*5*,*6*). Many publications provide insight on a given IFI and its trends in specific risk groups, but the overall burden of illness associated with IFI and its trends at a country level have not been described (*7*–*10*). To describe the epidemiology and trends of IFIs and to better identify public health priorities (e.g., surveillance, research, prevention strategies), we analyzed the national hospital discharge database of France, Programme de Médicalisation du Système d’Information, spanning 2001–2010.

## Materials and Methods

The national hospital database covers >95% of the country's hospitals (*11*). An anonymous subset of this database can be made available for epidemiologic studies without need for ethical approval or consent of patients, according to legislation by the government of France. A unique anonymous patient identifier enables distinction among first and subsequent hospital admissions. Information filed at discharge includes the major cause of admission and associated diseases, coded according to the International Classification of Diseases, Tenth Revision, the medical and surgical procedures performed, and the outcome including transfer, discharge, or death. Details on the data source, case definitions, and methods used are available in Technical Appendix 1.

Records of all hospital stays for which an IFI was recorded as the principal cause of admission or as a related disease were extracted from the national database for the period of January 2001 through December 2010. Records of the 5 most frequent IFIs were retained for this analysis. To facilitate comparisons with published studies, we restricted the study of invasive candidiasis to candidemia (i.e*.*, excluding *Candida* endocarditis and meningitis), and invasive aspergillosis (IA) included pulmonary and disseminated cases. All cryptococcosis cases were included. Gastrointestinal mucormycoses were excluded because results of a previous study showed that cases were mostly identified on the basis of false-positive test findings (*12*). Finally, codes corresponding to “pneumocystosis” or “HIV infection resulting in pneumocystosis” were designated as Pjp only if pneumonia was associated. We excluded rare IFIs (<40 cases per year each) and endemic mycoses (histoplasmosis, blastomycosis, coccidioidomycosis, sporotrichosis). Analysis focused on metropolitan areas of France, excluding overseas territories.

After checking for multiple stays and inconsistent records within and between hospitals, we retained “incident cases,” i.e., unique stays and first admissions. To reduce underreporting bias, we ensured that a risk factor that occurred during subsequent stays was integrated into the incident record (e.g., a diagnosis of diabetes recorded after a patient's transfer from a first- to a third–level hospital). Similarly, in-hospital fatality rates were estimated from the cumulative stays.

To describe risk factors associated with IFIs, we selected 9 conditions on the basis of expert opinion and published studies on the epidemiology of IFI. Considering the high diversity of conditions, and to provide a description relevant for clinical practice and health policy makers, we used hierarchical ranking to assign 1 risk factor per patient. Given that the preponderant risk factors differ among IFIs, IFIs were divided into 2 groups. In the first group, which included candidemia, IA, and mucormycosis, risk-factor ranking started with hematologic malignancies (HM, including by priority order, HM associated with hematologic stem cell transplantation [HSCT], HM not associated with HSCT but with neutropenia, and HM with none of the above factors). The following illnesses and conditions were subsequent risk factors in the first group: HIV/AIDS, solid organ transplantations, solid tumors, systemic inflammatory diseases (including inflammatory bowel diseases, sarcoidosis, rheumatoid arthritis, and systemic lupus or vasculitis of other origins), diabetes mellitus, chronic respiratory diseases (including chronic obstructive pulmonary diseases, asthma, and cystic fibrosis), chronic renal failure, and a group labeled “other diseases” that includes acute renal failure, liver cirrhosis, morbid obesity, acute or chronic pancreatitis, and severe burns. Thus, a case-patient with HM and diabetes was recorded as HM. For the second IFI group (Pjp and cryptococcosis), HIV/AIDS was the first risk factor, followed by other risk factors as described above. For all case-patients with IFI, additional risk factors were explored without hierarchical ranking: a stay in an intensive care unit; surgery; and extreme age, defined as neonates (≤28 days of age) and elderly adults (≥80 years of age). Because of lack of precise coding for several risk factors until 2003, only those documented during the 2004–2010 period were analyzed.

We expressed annual incidence rates among the general population, by gender and age groups, as cases per 100,000 population, using data from the 1999 national population census and its updates. We also analyzed trends in groups with selected risk factors, for which the respective denominators were available from routine surveillance data or from prevalence estimates, as detailed in Technical Appendix 1: patients with HM, HIV/AIDS, solid tumors, chronic renal failure, diabetes, and HSCT recipients. In these specific populations, we estimated the annual proportion of each IFI using the given risk factor per 100,000 population (2004–2010). Finally, we used an age-polynomial fractional logistic regression (*13*) to calculate age- and sex-adjusted risk for death categorized by risk factor, and analyzed each risk factor independently from the others without hierarchical ranking. We applied Fisher or χ^2^ tests to compare groups, and a Poisson regression to assess trends, considering p≤0.05 as significant, using Stata version 11.2 (StataCorp LP, College Station, TX, USA) software for all calculations.

## Results

### Characteristics of Case-Patients, 2001–2010

There were 35,876 cases of IFI registered in metropolitan France during 2001–2010 (Table 1). Candidemia accounted for the highest proportion of cases (43.4%); the next most frequently identified diseases were Pjp (26.1%), IA (23.9%), cryptococcosis (5.2%), and mucormycosis (1.5%). The overall incidence was 5.9/100,000 population per year. A total of 9,889 (27.6%) case-patients died while in a hospital. Candidemia and IA accounted for 87.6% of these deaths. Male patients predominated in all IFIs (64.0%), especially in Pjp and cryptococcosis (>70%). The mean age was 54.7 years (range 0–107 years). Gender and age characteristics of case-patients and of those who died differed according to the IFI. Details are provided in online Technical Appendix 2, Table 1. Incidence and fatality rates of candidemia and IA were particularly high in patients ≥60 years of age, and male patients predominated in all age groups, except in those >80 years of age. Case-patients in extreme age groups included 185 neonates (mainly with candidemia: 174 cases, 61.5% male patients, specific incidence 2.2/100,000 population) and 3,030 adults >80 years of age (2,283 with candidemia: 50.5% male, incidence 8.1/10^5^). Among case-patients with Pjp and cryptococcosis, the proportion of male case-patients was higher among HIV-infected persons than in non–HIV-infected persons (Pjp 74.0% vs. 62.2%; cryptococcosis 77.9% vs. 62.3%, respectively). 

**Table 1:** Cases of invasive fungal infection and attributable deaths in metropolitan France by disease, sex, and age, 2001–2010*

Infections	No. case-patients	Male sex, %	Age, y, median (IQR)	Illness incidence (95% CI)†	Fatality rate, % (95% CI)
Candidemia					
Cases	15,559	58.8	64 (51–75)	2.5 (2.1–2.9)	
Deaths	6,217	60.0	69 (56–77)		40.0 (38.7–42.0)
Pneumocystis pneumonia					
Cases	9,365	71.3	44 (37–55)	1.5 (1.2–1.9)	
Deaths	862	71.9	58 (43–70)		9.2 (7.6–12.4)
Invasive aspergillosis‡					
Cases	8,563	63.9	58 (45–68)	1.4 (1.2–1.6)	
Deaths	2,443	66.7	61 (49–71)		28.5 (26.9–30.5)
Cryptococcosis‡					
Cases	1,859	72.3	43 (36–55)	0.3 (0.2–0.4)	
Deaths	278	73.4	49 (39–65)		15.0 (13.2–17.9)
Mucormycosis‡					
Cases	530	57.7	58 (43–71)	0.09 (0.07–0.1)	
Deaths	89	62.9	57 (44–67)		16.8 (11.3–20.2)
Total					
Cases	35,876	64.0	56 (42–70)	5.9 (5.5–6.3)	
Deaths	9,889	63.1	65 (53–75)		27.6 (25.3–29.7)
*A total of 197 *Candida*-related endocarditis and 10 meningitis cases were excluded from analysis. IQR, interquartile range. †Incidence expressed as number of cases per 100,000 population per year (averaged over 10 y) ‡Invasive aspergillosis includes 91.7% pulmonary and 8.3% disseminated cases. Cryptococcosis includes 63.8% cerebral or disseminated forms; 13.2% pulmonary, cutaneous, or bone localizations; and 23.0% unspecified; forms. Mucormycosis includes 50.9% pulmonary, rhinocerebral and disseminated forms; 16.9% cutaneous forms; and 32.1% unspecified forms.					

The highest incidences of Pjp and cryptococcosis were observed among persons 30–59 years of age with AIDS and among those ≥60 years of age who were not infected with HIV (p<0.001 for each IFI). For these 2 IFIs, the fatality rate was lower in HIV-infected patients than in non-HIV-infected patients (Pjp 5.7% vs. 21.5%, p<0.001; cryptococcosis 13.4% vs. 17.9%, p<0.009).

### Trends in the General Population, 2001–2010

**Figure 1:**
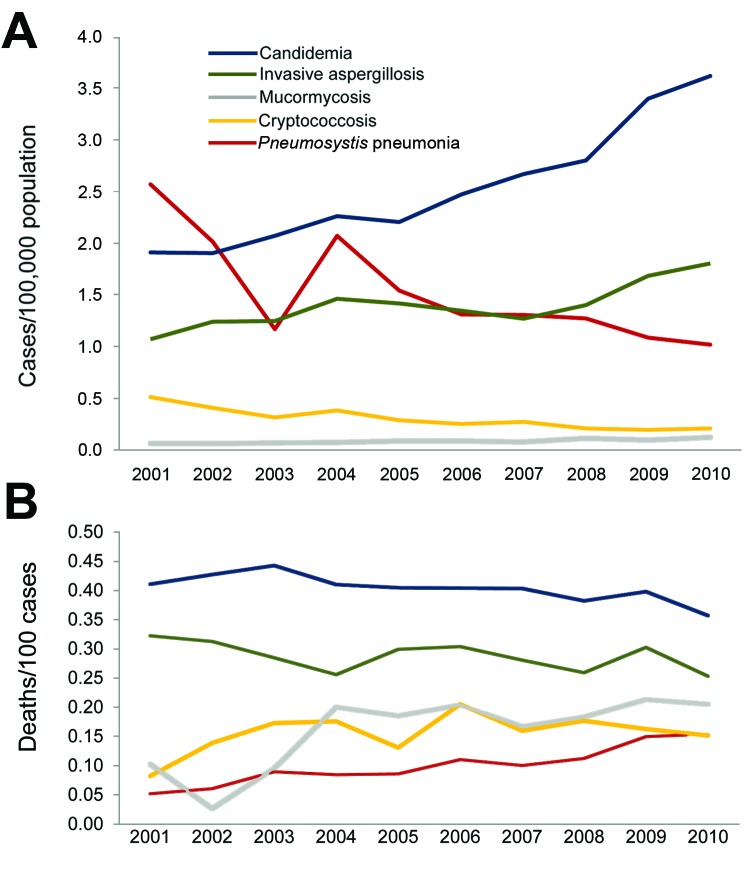
A) Trends in the incidence of invasive fungal infections in France, 2001–2010. The incidence increased (p<0.001) for candidemia, invasive aspergillosis, and mucormycosis, but decreased for cryptococcosis and pneumocystosis (Poisson's regression). B) Trends in the fatality rate by invasive fungal infections during 2001–2010. Fatality rates decreased for candidemia (p<0.001) and invasive aspergillosis (p = 0.04), but increased for mucormycosis (p = 0.03), pneumocystosis (p<0.001), and cryptococcosis (p = 0.03).

The incidence of IFI increased by 1.5% per year and that of deaths by 2.9% per year (p<0.001 each) over the 10-year period of observation. Specifically, the incidence of candidemia, IA, and mucormycosis increased by 7.8%, 4.4%, and 7.3% per year, respectively (p<0.001 each). The fatality rate decreased by 1.6% per year (p<0.001) among persons with candidemia and 1.4% per year (p = 0.04) among those with IA, but increased by 9.3% per year (p = 0.03) for those with mucormycosis. Regarding Pjp and cryptococcosis, incidence decreased by 8.6% and 9.8% per year (p<0.001 each), and the fatality rate increased by 11.7% (p<0.001) and 4.7% (p = 0.03) per year, respectively (Figure 1, panels A, B; Tables 2, 3). However, trends differed according to HIV status (Technical Appendix 2, Figure 1); incidence of both IFIs decreased among HIV-infected patients (Pjp −14.3%; cryptococcosis −14.9% per year, p<0.001 each), and Pjp increased in non–HIV-infected patients (+13.3% per year, p<0.001); there was no significant trend for cryptococcosis in non–HIV-infected patients. The fatality rate trend was only significant for HIV-associated Pjp (+5.6% per year, p = 0.001).

**Table 2:** Cases of invasive fungal infections per 100,000 population, metropolitan France, 2001–2010

Disease	2001	2002	2003	2004	2005	2006	2007	2008	2009	2010
*Pneumocystis* pneumonia	2.6	2.0	1.2	2.1	1.5	1.3	1.3	1.3	1.1	1.0
Candidemia	1.9	1.9	2.1	2.3	2.2	2.5	2.7	2.8	3.4	3.6
Invasive aspergillosis	1.1	1.2	1.3	1.5	1.4	1.4	1.3	1.4	1.7	1.8
Cryptococcosis	0.5	0.4	0.3	0.4	0.3	0.3	0.3	0.2	0.2	0.2
Mucormycosis	0.07	0.06	0.07	0.07	0.09	0.09	0.08	0.11	0.10	0.12

**Table 3:** Deaths attributed to invasive fungal infections per 100,000 cases, metropolitan France, 2001–2010

Disease	2001	2002	2003	2004	2005	2006	2007	2008	2009	2010
Candidemia	0.41	0.43	0.44	0.41	0.40	0.40	0.40	0.38	0.40	0.36
Invasive aspergillosis	0.32	0.31	0.28	0.26	0.30	0.30	0.28	0.26	0.30	0.25
Mucormycosis	0.10	0.03	0.10	0.20	0.19	0.20	0.17	0.18	0.21	0.21
Cryptococcosis	0.08	0.14	0.17	0.18	0.13	0.21	0.16	0.18	0.16	0.15
*Pneumocystis* pneumonia	0.05	0.06	0.09	0.08	0.09	0.11	0.10	0.11	0.15	0.15

### Risk Factor Distribution and Trends in the General Population, 2004–2010

We studied risk factors among 25,933 IFI case-patients identified during the 2004–2010 period. Candidemia remained the most frequent IFI (46.4%) followed by IA (24.8%) and Pjp (22.9%). The distribution of risk factors differed for each IFI (Technical Appendix 2, Table 2). Solid tumors were mainly found in patients with candidemia (30.6%), HM in those with IA and mucormycosis (54.3% and 34.8%, respectively), and HIV/AIDS in those with Pjp and cryptococcosis (>55% each). The incidence of candidemia, IA, and mucormycosis in patients with HM (especially with neutropenia) increased significantly, as did the incidence of candidemia and IA in solid organ transplant recipients, and patients with solid tumors or chronic renal failure. The incidence of Pjp decreased in patients with HM and increased in patients with solid organ transplants, solid tumors, and chronic renal failure.

### IFI Trends in Specific Risk Groups, 2004–2010

**Figure 2:**
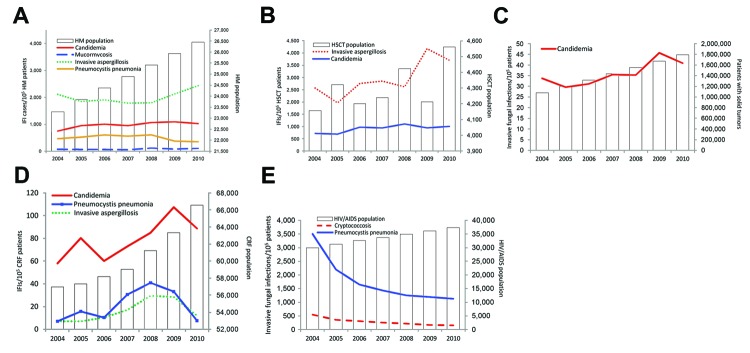
A) Invasive fungal infections in patients with hematologic malignancies (HM) in France, 2004–2010. The case count continuously increased (p<0.001) over the period. Candidemia increased from 751.4 to 1,028.2 cases (+4.3%, p = 0.001), invasive aspergillosis (IA) from 2,112.4 to 2,434.2 cases (+2.7%, p = 0.002), and mucormycosis from 73.0 to 105.8 cases (+8.7%, p = 0.05) per 100,000 patients per year. Inversely, the incidence of *Pneumocystis jirovecii* pneumonia (Pjp) decreased from 468.0 to 351.5 cases/100,000 patients/year (−4.4%, p = 0.006). B) In HSCT recipients (average 4,300 cases per year, no significant trend), candidemia increased from 721.5 to 1008.6 cases (+6.0%, p = 0.05) and invasive aspergillosis from 2,573.4 to 3,705.3 cases (+9.8%, p<0.001) per 100,000 HM patients per year. C) The number of patients with solid tumors continuously increased (p<0.001), and candidemia increased among those patients from 33.7 to 40.9 cases/100,000 patients/year (+6.2%, p<0.001). D) The number of patients with chronic renal failure continuously increased (p<0.001). Candidemia increased from 57.9 to 88.6 cases/100,000 patients/year (+8.1%), IA from 7.0 to 12.0 cases/100,000 patients/year (+18.4%, p = 0.007), and Pjp increased with a peak during 2007–2008 (+11.1%, p = 0.052). E) In the HIV/AIDS population (increase p<0.001), incidence of Pjp and cryptococcosis decreased by −17.9% and −19.0%, respectively (p<0.001). HSCT, hematologic stem cell transplant.

We estimated trends from the annual proportion of risk factor–associated IFIs in the corresponding risk population. Only statistically significant trends are shown in Figure 2. In the general population, the number of patients with HM, solid organ transplantations, chronic renal failure, HIV/AIDS, and diabetes substantially increased over time, and the population of HSCT recipients remained unchanged. In patients with HM, there was a statistically significant increase of candidemia, IA, and mucormycosis, and a decrease of Pjp (Figure 2, panel A). In HSCT recipients, candidemia and IA increased (Figure 2, panel B).

During the study period, candidemia increased among patients who had solid tumors (Figure 2, panel C). Among patients with chronic renal failure, the incidence of candidemia, IA, and Pjp increased (Figure 2, panel D). Among patients with HIV/AIDS, the incidence of Pjp and cryptococcosis decreased (Figure 2, panel E). There was no substantial trend among patients with diabetes (data not shown).

### Odds Ratio of Death by Risk Factors, 2004–2010

We assessed the risk for death associated with each risk factor by logistic regression, considering each factor independently and expressed as an odds ratio for death; except for age, significant results are shown in Technical Appendix 2, Table 3. The risk for death was lower in female patients with IA, but did not differ by sex for other infections. The role of age varied according to the IFI type; for instance, in-hospital fatality rates increased in persons >20 years of age who had candidemia and Pjp, and in those >70 years who had IA. HM represented a substantial risk factor for death in patients with candidemia, IA, mucormycosis, and in non-HIV cryptococcosis. Solid tumors were a substantial risk factor for death in patients with candidemia, IA, and Pjp, regardless of HIV status. Cirrhosis and acute renal failure were also substantial risk factors for death in patients with candidemia, IA, and non-HIV Pjp and cryptococcosis. Hospitalization in an intensive care unit was associated with a higher risk for death among patients with all IFIs except candidemia. Inversely, chronic renal failure decreased the risk for death among those with IA or Pjp, respiratory diseases decreased the risk in patients with IA, and surgical procedures decreased the risk for those with candidemia.

## Discussion

This nationwide study provides evidence that ≈3,600 patients have IFI each year in France, of whom 28% die. The incidence of candidemia, IA, mucormycosis, and non-HIV Pjp has increased over the last decade, predicting a protracted trend over the coming years.

Studies on the epidemiology of the 5 predominant IFIs have reached conflicting results, depending on the IFI studied (most studies focused on a single IFI), the study design, and source of data (active surveillance system, cohorts, multicentric or monocentric, laboratory-based diagnosis, hospital discharge data), the population of interest (neutropenic patients, HM, HSCT and solid organ transplant recipients), and the practices regarding antifungal agents use (prophylactic, empiric, preemptive, or curative therapy). Here, we analyzed the hospital dataset at a country level, covering all persons who were admitted to hospitals over a period of 10 years, regardless of age or underlying conditions. We included those with illness caused by IFIs that have straightforward diagnostic criteria (candidemia, cryptococcosis) or well-characterized clinical entities (pulmonary or disseminated IA, pulmonary Pjp), as well as mucormycosis, for which we previously validated the accuracy of diagnostic coding in the hospital national database (*14*,*15*). Despite potential bias in the precise classification of cases, particularly for mold infections, and other limitations of administrative datasets that have been previously discussed (*12*,*14*,*16*), several points validate the findings obtained through this large database. The predominance of candidemia and IA has been described in other studies of a variety of IFIs in the general population or in other groups (*7*,*9*,*17*). For candidemia, the incidence and trends we estimated are comparable to many other, although smaller scale, population-based studies from Europe and North America (*18*–*22*). For IA in France, we observed a lower incidence and higher mortality rate than were found by Dasbach et al. in their analysis of US hospital discharge data . (*23*). The differences may be explained by the researchers’ use of the International Classification of Diseases, Ninth Revision case definitions in that study, which would impair the comparison of invasive and noninvasive forms.

The decreasing incidence of Pjp and cryptococcosis was expected after the advent of active antiretroviral therapy (*5*,*6*,*24*,*25*). However, we observed some noteworthy changes: Pjp incidence in non-HIV–infected patients has currently reached the levels observed in HIV-infected patients, as observed in the United Kingdom during the same period (*26*); incidence of cryptococcosis is also increasing in the seronegative population, and the mortality rate of both IFIs among non-HIV–infected patients is higher than among HIV-infected patients.

Most risk factors described in this study are well known in clinical practice. The major risk factors for candidemia, IA, and mucormycosis, i.e., HM, HSCT, and solid tumors, are described in many studies, such as those by the Transplant Associated Infections Surveillance Network, known as TRANSNET, and Prospective Antifungal Therapy Alliance, known as PATH (*3*,*27*–*29*), albeit sometimes reported as differently distributed. The hierarchical ranking process used here may have influenced the risk factor distribution, underestimating some conditions. Most studies of risk factors are performed on the basis of cohorts of cases in referral centers where a large number of high-risk patients are recruited, whereas in our population-based approach, we used a national dataset covering all levels of care, thus selecting a wider range of underlying conditions, including those less commonly recognized as risk factors. As a result, we documented substantial increases of candidemia, IA, and Pjp in patients with chronic renal failure, suggesting that the increase is not uniquely caused by the growing number of persons at risk (Figure 2). The growing number and longer survival of patients with protracted immunosuppression beyond traditional hematology patients, transplant patients, and HIV/AIDS populations are major challenges. The fact that 2 IFIs that are frequently associated with health care settings (candidemia and IA) are still on the rise despite existing infection control recommendations is of specific concern (*30*).

Hospital data are not collected for clinical research purposes. Thus, it is very hazardous to explain the trends on the basis of our limited observations. Specific analyses should be encouraged, aiming at better understanding the role of comorbid conditions in the occurrence of IFI (e.g., chronic renal failure) or the effect of the improved overall survival of patients, even those who are immunocompromised.

Another noteworthy finding of this study is that the risk for death was altered by factors that were not frequently documented before. For instance, cirrhosis was found in 1.3% of all patients with IFIs but was an independent risk factor for death among all except those with mucormycosis, suggesting underrecognition of IFIs in such populations, possibly leading to delayed prevention or treatment. Similarly, patients with HM showed an increased risk for death when cryptococcosis was also diagnosed, as did those with cirrhosis and acute renal failure, which suggest that specific attention should be paid to patients with these conditions; this could modify their clinical management.

This population-based study has limitations. The increase in IFIs observed parallels a better awareness of clinicians and microbiologists of the threat of IFIs in at-risk populations, improving the sensitivity of the hospital-based dataset. The availability of a broader antifungal drug armamentarium and efficient treatment could have the paradoxical effect of improving the prevention of IFI for selected groups of at-risk patients, thus lowering the population of infected patients. We report trends and risk factors for invasive mycosis in France. Hence, our findings may not apply to other countries with different endemic mycoses, population structures, and health care systems. Our observations are based on hospital discharge coding, which is subject to many biases, including misdiagnosis and incorrect coding. More notably, the advent of new diagnostic tools for the detection of many invasive mycoses may have affected our ability to diagnose these diseases over the study period, which may have had a substantial impact on the temporal trends observed.

Nevertheless, this large-scale study provides benchmarking data on the current burden of illness of major IFIs and shows the effects of disease trends and death rates spanning a decade in a Western European country. The need for baseline data was recently highlighted (*10*). Our data provide complementary information to specific studies or investigations linked to outbreaks (*31*,*32*). IFIs in this study occurred among a broad spectrum of patients and the fatality rate was high; clinicians should be made aware of risk factors, signs, and symptoms. Beyond the specific issues addressed by our study, such as the identification and management of patients in potentially under-recognized risk groups, the expected consequences of the increasing incidence of IFIs should be anticipated in terms of hospital and laboratory workload, antifungal use, and need for new systemic antifungal drugs and strategies (*33*). The development of epidemiologic studies is also of specific concern to clarify the determinants of the trends and identify effective interventions that can reduce deaths and the general public health burden of illness. These questions should be addressed jointly by clinicians and public health authorities at national and international levels.

Technical Appendix 1 Methods: the French hospital information system, data sources, case definitions, and risk factors for invasive fungal infections, France, 2001–2010.

Technical Appendix 2 Incidence and mortality rates, risk factors and trends, demographics, and distribution of invasive fungal infections, France, 2001–2010.
