# Supplementary material for: Population-Based Analysis of Invasive Fungal Infections, France, 2001–2010
Source: Emerg Infect Dis. 2014 Jul;20(7):1149–55. doi: 10.3201/eid2007.140087 (PMC4073874; doi:10.3201/eid2007.140087)
Supplement: Technical Appendix 1 — Methods: the French hospital information system, data sources, case definitions, and risk factors for invasive fungal infections, France, 2001–2010. [file 14-0087-Techapp-s1.pdf]

Article DOI: 10.3201/eid2007.140087

Population-Based Analysis of Invasive Fungal Infections, France, 2001–2010

Technical Appendix 1: Methods

1. The French Hospital Information System

1a. The Database

The national hospital discharge database *PMSI (Programme de médicalisation des systèmes d'information*) systematically collects information for any new admission: anonymous patient identifier, the hospital code, the patient's gender, age, residence area, main cause of admission, other medical conditions reported during a stay (>20 entries), acts performed during the stay, duration of stay and mode of discharge including transfer or death when death occurs at hospital. The combination of diseases and acts provides a "discharge summary code" which infers the cost of each stay, provides useful information on major surgery, and allows to check the consistency of collected information (only available from 2004 onwards). This discharge summary code is adapted from the American DRG classification.

The 10th international diseases classification (ICD10) codes are used. These codes were stable over the study period, except for a few variables such as body mass index thresholds for morbid obesity, introduced in 2009.

Medical and surgical acts performed during each stay are coded using the national health insurance classification, which evolved over time. For instance, autologous vs. allogenic hematopoietic stem cell transplant (HSCT) recipients were not distinguished before 2003, and were not systematically differentiated after this period.

1b. Selection of Incident Cases

The patient anonymous identifier, available since 2003, was used to distinguish first admissions from re-hospitalizations. For previous years another identifier was created by chaining variables "year of birth" (derived from the patient's age and date of admission), "gender" and "residence zip code." Duplicates were checked within and between hospitals.

Cases detected once over the period were "unique cases", those detected >1 time were "newly admitted case” at first occurrence and “re-admitted” at subsequent occurrence(s).

For re-admitted patients, delays between subsequent stays were estimated from admission and discharge dates. By convention, a case with subsequent stays was defined as a single episode if delay was ≤180 days and as a new episode after this delay. As the proportion of new episodes was very low (<5% of each selected IFI, <8% for *Pneumocystis jiroveci* pneumonia), we did not retain these new episodes in the final analysis

1c. Finalisation of the Database with 5 Selected IFIs

The dataset was first created for each selected IFI, including the identification of unique and first admissions (incident cases), the hierarchisation of risk factors and the reporting of death.

In the resulting merged dataset including the five IFIs, when one IFI was reported as principal diagnosis and another one was reported as “associated disease”, priority was given to the first one. When two IFIs were reported as “associated diseases”, priority was given to the rarest one, in order to reduce the under-estimation bias for these rare infections. For instance, a case was considered as mucormycosis when mucormycosis and candidemia were recorded. This process only concerned <1% of the overall dataset.

2. Estimations of Incidence and Trends: Data Sources

•Annual incidence rates by gender and age groups were estimated using the 1999 national population census and its updates, available at the national public health institute (InVS). For neonates (0-28 days) we hypothesised their number was equal to the number of live births.

•Incidence in specific groups was estimated for the following conditions for which annual numbers were available:

- Patients with hematological malignancies (HM): data provided by Réseau Francim (http://lesdonnees.e-cancer.fr/information/8-base-commune-des-registres-de-cancers.html)
- HIV/AIDS: subject to mandatory notification. Data retrieved from the routine surveillance system, available at InVS (http://www.invs.sante.fr)

- Solid tumors patients: we used as a proxy, the number of cancer patients regularly followed up in each hospital. Data available at the Ministry of Health's division of statistics website (http://www.drees.sante.gouv.fr/statistique-annuelle-des-etablissements-sae,6506.html)
- Chronic renal failure patients: data provided by National agency in charge: Agence de la Biomédecine (http://www.agence-biomedecine.fr/agence/english.html)
- Diabetes: prevalence data on treated diabetic patients and point estimates for all cases obtained from two studies (*1*,*2*)
- Hematopoietic stem cell transplant (HSCT) recipients: data provided by National agency in charge: Agence de la Biomédecine (http://www.agence-biomedecine.fr/agence/english.html)

3. Case Definitions for Selected IFIs: ICD-10 codes

Invasive Aspergillosis: Invasive pulmonary (*B44.0)* or Disseminated aspergillosis (*B44.7).*

Candidemia: Candidal sepsis (*B37.7).*

Candidal meningitis (B*37.5)* and endocarditis (*B37.6)* were excluded from analysis to allow international comparisons

Cryptococcosis: All codes *B45*: Pulmonary (*B45.0),* Cerebral incl. meningitis (*B45.1),* Cutaneous (*B45.2),* Osseous (*B45.3),* Disseminated (*B45.7),* Other (*B45.8)* or Unspecified forms (*B45.9).*

***Pneumocystis* pneumonia:** Pneumonia due to *Pneumocystis carinii* / P. jiroveci (B59) or HIV disease resulting in *Pneumocystis jirovecii* pneumonia (*B20.6)* *and* Confirmed pneumonia

Zygomycosis/mucormycosis: All *B46* except gastrointestinal (*B46.2).* All other codes included i.e. Pulmonary (*B46.0),* Rhinocerebral (*B46.1),* Cutaneous/subcutaneous (*B46.3),* Disseminated (generalized) (*B46.4) or* Unspecified, (*B46.5*; *B46.8*; *B46.9).*

Gastro-intestinal forms were excluded based on the retrospective study ("*RetroZygo"*) among patients diagnosed in metropolitan France from 2005 to 2007 (*3*,*4*), in which cases recorded in the *PMSI* were identified and discussed with the medical teams. The vast majority of gastro-intestinal cases were secondarily excluded as false positives.

4. Case Definitions for Associated Diseases (Risk Factors for IFI)

We used ICD-10 codes, as well as the codes from the National health insurance classification (*NHIC*) which provided acts performed during a stay (surgical acts, transplantations)**.**

2.1. Hematological Malignancies (HM)

ICD-10: Acute lymphoid or myeloid leukaemia: C91; C92; C93. Hodgkin disease: C81. Follicular lymphoma: C82. Non-follicular lymphoma: C83. Mature T/NK-cell lymphoma: C84. Other and unspecified types of non-Hodgkin lymphoma: C85; C88. Multiple myeloma and malignant plasma cell neoplasms: C90. Other leukaemias of specified cell type: C94. Leukaemia of unspecified cell type: C95. Other and unspecified malignant neoplasms of lymphoid, hematopoietic and related tissue: C96. Myelodysplastic syndromes: D46. Other neoplasms of uncertain or unknown behaviour of lymphoid, hematopoietic and related tissue: D47.

NHIC acts: Chemotherapy for acute leukemias, lymphomas, other tumours or myeloproliferative disorders.

2.1.b. HM with Hematological stem cell transplantation

ICD-10: Bone marrow transplantation, Z94.8 including autologous (Z94.8.00) and allogenic (Z94.8.01) transplantations. Bone-marrow transplant rejection (T86.0).

NHIC acts: Autologous or allogenic stem cell transplantation. Intraveneous injection of cellular therapy products.

2.1.c. HM with neutropenia or medullar aplasia

ICD-10: Acquired pure red cell aplasia, D60. Other aplastic anaemia, D61. Agranulocytosis & functional disorders of polymorphonuclear neutrophils, D70; D71.

2.2. Non-hematological Immunosuppressions

2.2.a. HIV-AIDS

ICD-10: Human immunodeficiency virus (HIV) disease resulting in infectious and parasitic diseases, B20. HIV disease resulting in malignant neoplasms, B21. HIV disease resulting in other specified diseases, B22. HIV disease resulting in other conditions, B23. Unspecified HIV disease, B24 (including AIDS-related complex). Asymptomatic HIV infection status

2.2.b. Solid organ transplantation / rejection (other than HSCT)

ICD-10: Failure and rejection of transplanted organs and tissues,T86. Transplanted organ and tissue status, Z94.

NHIC: Heart, liver, lung, pancreas and renal tranplantations (NHIC).

2.2.c. Solid organ tumours

ICD-10: Malignant neoplasms, stated or presumed to be primary, of specified sites, except of lymphoid, hematopoietic and related tissue, C00 to C75. Malignant neoplasms of ill-defined, secondary and unspecified sites, C76 to C80.

2.3. Systemic Inflammatory Diseases

ICD-10: Crohn disease, K50. Sarcoidosis, D86. Rheumatoid arthritis, M05 (incl. M05.0; M05.1; M05.3; M05.8; M05.9) & M06. Lupus and vascularitis, M05.2; L930; M30; M31; M32

2.4. Diabetes Mellitus

ICD-10: Insulin-dependent diabetes mellitus, E10. Non-insulin dependent diabetes mellitus, E11. Malnutrition-related diabetes mellitus, E12. Other specified diabetes mellitus, E13. Unspecified diabetes mellitus, E14. Diabetes mellitus in pregnancy, O24

2.5. Chronic Respiratory Diseases

ICD-10: Chronic obstructive pulmonary disease (COPB): J44 (incl. J44.0 ; J44.1;J44.8;J44.9). Asthma, J45. Cystic fibrosis, E84.

2.6. Chronic Renal Failure

ICD-10: Chronic kidney disease, N18 including chronic renal failure, chronic uraemia and diffuse sclerosing glomerulonephritis. Congenital renal failure of the newborn, *P96.0*. Hypertensive renal disease with renal failure, *I12.0*. Hypertensive heart and renal disease with renal failure, *I13.1*. Dependence on renal dialysis, *Z99.2*.

NHIC: Renal failure with dialysis or dialysis sessions for chronic renal failure (*NHIC*)

2.7. Acute Renal Failure

ICD-10: Acute renal failure, *N17.*Post-procedural renal failure, *N99.0*. Renal failure following abortion and ectopic and molar pregnancy, *O08.4*. Postpartum acute renal failure, *O90.4*.

NHIC: Acute renal failure without dialysis or dialysis session for acute renal failure.

2.8. Morbid Obesity

ICD-10: Obesity due to excess calories with BMI [40-49kg/m²], *E66.01*. Obesity due to excess calories, BMI ≥ 50kg/m², *E66.02*. Drug-induced obesity, BMI [40-49kg/m²], *E66.11*. Drug-induced obesity, BMI ≥ 50kg/m², *E66.12*. Extreme obesity with alveolar hypoventilation, BMI [40-49kg/m²], *E66.21*. Extreme obesity with alveolar hypoventilation, BMI ≥ 50 kg/m², *E66.22*. Other obesities, BMI ≥ 40kg/ m², *E66.81; E66.82*.

NHIC: surgical treatments for morbid obesities (bypass, other acts).

2.9. Liver Cirrhosis

ICD-10: Alcoholic liver disease, *K70.0*. Alcoholic cirrhosis of liver, *K70.3*. Toxic liver disease with fibrosis and cirrhosis of liver, *K71.7*. Fibrosis and cirrhosis of liver, *K74* (incl. K74.3 - K74.6)

2.10. Pancreatitis (acute/chronic)

ICD-10: Acute pancreatitis, *K85*. Other diseases of pancreas, *K86* (incl. K86.0-K86.1)

2.11. Burns

ICD-10: Third degree burn: head and neck *T20.3*, trunk *T21.3*, shoulder and upper limbs *T22.3*, wrist and hand *T23.3*, hip and lower limb *T24.3*, ankle and foot *T25.3*. Burns of multiple regions, at least one burn of third degree mentioned, *T29.3*. Burn and corrosion, body region unspecified, third degree *T30.3*. Burns classified according to extent of body surface involved, *T31.1-31.9*

2.12. Surgery

Discharge summary codes: Head-neck-thoracic surgery, Abdomen-urinary tract and gynaecological surgery,and musculo-skeletal surgery

2.13. Intensive Care

ICD-10: Dependence on respirator, *Z99.1*.

Summary code: Injury severity score (*ISS)>24.*

References

1. Bonaldi C, Vernay M, Roudier C, Salanave B, Oleko A, Malon A, et al. A first national prevalence estimate of diagnosed and undiagnosed diabetes in France in 18- to 74-year-old individuals: the French Nutrition and Health Survey 2006/2007. Diabet Med. 2011;28:583–9. PubMed http://dx.doi.org/10.1111/j.1464-5491.2011.03250.x

2. Ricci P, Blotiere PO, Weill A, Simon D, Tuppin P, Ricordeau P, et al. Diabète traité: quelles évolutions entre 2000 et 2009 en France? Bull Epid Hebd 2010;425-31.

3. Bitar D, Morizot G, Vancauteren D, Dannaoui E, Lanternier F, Lortholary O, et al. Estimating the burden of mucormycosis infections in France (2005–2007) through a capture-recapture method on laboratory and administrative data. Rev Epidemiol Sante Publique. 2012;60:383–7. PubMed http://dx.doi.org/10.1016/j.respe.2012.03.007

4. Lanternier F, Dannaoui E, Morizot G, Elie C, Garcia-Hermoso D, Huerre M, et al. A global analysis of mucormycosis in France: the RetroZygo Study (2005–2007). Clin Infect Dis. 2012;54(Suppl 1):S35–43. PubMed http://dx.doi.org/10.1093/cid/cir880
