# Supplementary material for: Population-Based Analysis of Invasive Fungal Infections, France, 2001–2010
Source: Emerg Infect Dis. 2014 Jul;20(7):1149–55. doi: 10.3201/eid2007.140087 (PMC4073874; doi:10.3201/eid2007.140087)
Supplement: Technical Appendix 2 — Incidence and mortality rates, risk factors and trends, demographics, and distribution of invasive fungal infections, France, 2001–2010. [file 14-0087-Techapp-s2.pdf]

Article DOI: 10.3201/eid2007.140087

Population-Based Analysis of Invasive Fungal Infections, France, 2001–2010

Technical Appendix 2: Distribution of and Trends in invasive fungal infections by demographics, risk groups, and risk factors; France, 2001-2010

Technical Appendix 2, Table 1. Incidence and fatality rate of invasive fungal infections averaged by invasive fungal type, age group, and gender, metropolitan France, 2001–2010*

	Candidemia			Invasive aspergillosis			Mucormycosis			*Pneumocystis* pneumonia						Cryptococcosis					
										HIV/AIDS			Non-HIV/AIDS			HIV/AIDS			Non-HIV/AIDS		
Age, days/years	Incid	Sex, M (%)	FR	Incid	Sex, M (%)	FR	Incid	Sex, M (%)	FR	Incid	Sex, M (%)	FR	Incid	Sex, M (%)	FR	Incid	Sex, M (%)	FR	Incid	Sex, M (%)	FR
0–29 d	2.2	61.5	0.25	0.1	57.1	0.57	0.03	50.0	0.00	NA	NA	NA	NA	NA	NA						
30 d–9 y	0.5	59.2	0.15	0.3	56.7	0.20	0.02	57.1	0.07	0.05	42.9	0.03	0.09	51.6	0.10	NA	NA	NA	0.03	68.4	0.05
10–19 y	0.3	57.5	0.18	0.5	64.7	0.20	0.03	57.1	0.19	0.04	27.3	0.03	0.04	60.6	0.09	0.01	37.5	0.25	0.02	50.0	0.06
20–29 y	0.6	51.8	0.21	0.6	54.2	0.24	0.05	64.1	0.18	0.49	53.2	0.03	0.10	57.3	0.11	0.16	54.5	0.09	0.06	51.0	0.08
30–39 y	1.0	58.2	0.21	0.8	57.4	0.22	0.05	62.5	0.08	2.80	68.5	0.05	0.15	53.6	0.07	0.50	75.7	0.12	0.08	71.8	0.06
40–49 y	1.7	56.2	0.32	1.3	63.9	0.25	0.08	58.8	0.24	3.11	78.6	0.05	0.24	58.2	0.14	0.51	82.7	0.15	0.09	75.3	0.08
50–59 y	3.4	62.0	0.37	2.4	66.1	0.26	0.13	61.5	0.20	1.56	82.6	0.06	0.53	64.4	0.16	0.19	86.8	0.16	0.14	66.7	0.18
60–69 y	5.8	64.7	0.42	3.6	66.9	0.31	0.18	64.0	0.23	0.71	77.8	0.13	1.00	68.1	0.24	0.08	88.9	0.16	0.23	60.9	0.21
70–79 y	8.1	58.6	0.47	3.1	65.2	0.37	0.16	56.6	0.09	0.25	67.8	0.22	1.02	59.2	0.31	0.01	100.0	0.33	0.23	65.4	0.30
80–89 y	8.6	52.0	0.52	1.8	58.3	0.34	0.23	38.2	0.09	0.03	37.5	0.25	0.56	65.4	0.36	NA	NA	NA	0.31	41.1	0.30
≥90 y	5.3	37.8	0.50	0.6	46.2	0.42	0.24	27.3	0.18	0.02	100	0.00	0.13	16.7	0.17	NA	NA	NA	0.15	42.9	0.00
Average	2.5	58.8	0.40	1.4	63.9	0.29	0.09	57.7	0.17	1.2	74.0	0.06	0.34	62.2	0.21	0.2	77.9	0.13	0.11	62.3	0.18

*Incid, incidence, FR, fatality rate, NA, not applicable to this age group.

Technical Appendix 2, Table 2. Invasive fungal infections in metropolitan France, 2004–2010: distribution of risk factors by type of IFI and evolution of incidence (trend) in the population*

	Candidemia				Invasive aspergillosis				Mucormycosis				*Pneumocystis* pneumonia				Cryptococcosis			
		Incidence trend				Incidence trend				Incidence trend				Incidence trend				Incidence trend		
Risk factors	No.	Evol.	%	p value	No.	Evol.	%	P value	No.	Evol.	%	P value	No.	Evol.	%	p value	No.	Evol.	%	pvalue
Hematologic malignancy	1,710	+	5.8	<0.001	3,496	+	4.2	<0.001	143	+	10.3	0.02	869	–	3.0	0.06	88	NA	NA	NA
with HSCT	276	+	6.4	0.04	890	+	10.3	<0.001	40	NA	NA	NA	115	NA	NA	NA	7	NA	NA	NA
with neutropenia	721	+	7.0	<0.001	1,540	+	8.0	<0.001	53	+	20.7	<0.001	203	NA	NA	NA	15	NA	NA	NA
with none of above	713	+	4.4	0.02	1,066	–	-5.4	<0.001	50	NA	NA	NA	551	–	5.8	0.004	66	NA	NA	NA
HIV/AIDS	142	NA	NA	NA	94	NA	NA	NA	15	NA	NA	NA	4,047	–	15.4	<0.001	656	–	16.4	<0.001
Solid organ transplant	190	+	7.3	0.05	231	+	12.5	<0.001	9	NA	NA	NA	108	+	13.1	0.01	29	NA	NA	NA
Solid tumor	3,683	+	14.6	<0.001	473	+	4.7	0.049	19	NA	NA	NA	227	+	9.9	0.005	35	NA	NA	NA
Systemic inflammatory diseases	178	+	8.3	0.04	140	NA	NA	NA	9	NA	NA	NA	106	NA	NA	NA	30	NA	NA	NA
Diabetes	1,123	+	8.3	<0.001	207	NA	NA	NA	68	NA	NA	NA	76	NA	NA	NA	51	NA	NA	NA
Chronic respiratory diseases	433	+	4.7	0.06	529	NA	NA	NA	13	NA	NA	NA	33	NA	NA	NA	17	NA	NA	NA
Chronic renal failure	336	+	10.3	<0.001	68	+	21.0	0.003	8	NA	NA	NA	88	+	13.4	0.02	20	NA	NA	NA
Other diseases†	1,751	+	6.4	<0.001	189	+	10.7	0.006	19	NA	NA	NA	78	+	22.0	<0.001	32	NA	NA	NA
Unspecified ‡	2,493	+	6.3	<0.001	1,006	+	ns	NA	108	NA	NA	NA	299	NA	NA	NA	161	NA	NA	NA
Total	12,039	+	9.2	<0.001	6,433	+	4.00	<0.001	411	+	7.6	0.003	5,931	–	10.2	<0.001	1,119	–	9.9%	p<0.001

*Evol.: evolution of incidence over the period (increase +, decrease -); NA, not applicable; no substantial evolution over the period; P = p value of trend; %: annual percentage of increase or decrease of incidence in the general population.
†Other diseases: the 1,751 patients with candidemias accounted for 15.8% of cirrhosis, <6% of morbid obesity and pancreatitis, and 84.6% acute renal failure (not exclusive). The 189 invasive aspergillosis case-patients accounted for 24.3% of cirrhosis and 87.8% acute renal failure. The 19 mucormycosis case-patients included 10.5% of cirrhosis and 63.2% acute renal failure.
†Unspecified: The 2,493 patients with candidemia required a stay in an intensive care unit (ICU) (841 cases; 6.9% of the total), surgical procedures (317 cases; 2.6%), extreme age with no other known factor (484 cases; 4.0%), and 851 cases (7.1%) with no specific risk factor. The 108 mucormycosis case-patients required a stay in ICU or surgical ward (20 cases;4.9%), extreme age (27 cases; 6.6%) and 61 cases (14.8%) with no specific risk factor. Data for the 299 patients with *Pneumocystis jirovecii* pneumonia required a stay in ICU or surgical procedures (56 cases; <1% of the total), extreme age with no other risk factor (<1%) and 226 cases (3.8%) with no specific risk factor. The 161 cryptococcosis cases required a stay in ICU or surgical ward (46 cases; 4.1%), extreme age (27 cases; 2.4%) and 95 cases (8.5%) with no specific risk factor.

Technical Appendix 2, Table 3. Age- and sex–adjusted odds ratio (OR) of death by invasive fungal infections and by significant (p<0.05) risk factor, metropolitan France, 2004–2010

Patient characteristics	Candidemia		Invasive Aspergillosis		Mucormycosis		Pneumocystis pneumonia, HIV/AIDS(-)		Cryptococcosis., HIV/AIDS(-)		Pneumocystis pneumonia, HIV/AIDS(+)		Cryptococcosis, HIV/AIDS(+)	
	OR	95% CI	OR	95% CI	OR	95% CI	OR	95% CI	OR	95% CI	OR	95% CI	OR	95% CI
Age, y														
<10	1	1.00-1.00	1	1.00-1.00	1	1.00-1.00	1	1.00-1.00	NA	NA	NA	NA	NA	NA
10–19	1,05	1.04-1.05	0,75	0.64-0.86	1,19	0.99-1.39	1,01	1.01-1.02	NA	NA	1	1.00-1.00	NA	NA
20–29	1,16	1.14-1.18	0,69	0.55-0.83	1,43	0.95-1.90	1,07	1.06-1.09	1	1.00-1.00	1,39	1.21-1.57	1	1.00-1.00
30–39	1,35	1.31-1.39	0,69	0.54-0.85	1,70	0.85-2.56	1,22	1.17-1.26	1,72	1.25-2.19	1,93	1.43-2.43	1,44	1.17-1.70
40–49	1,64	1.56-1.71	0,75	0.57-0.93	2,03	0.67-3.40	1,52	1.39-1.64	2,47	1.35-3.59	2,68	1.64-3.73	2,06	1.31-2.81
50–59	2,07	1.93-2.22	0,87	0.66-1.09	2,43	0.39-4.47	2,14	1.83-2.45	3,55	1.29-5.80	3,73	1.79-5.66	2,96	1.35-4.57
60–69	2,74	2.48-3.00	1,11	0.83-1.39	2,90	-0.02-5.82	3,50	2.66-4.34	5,09	0.93-9.26	5,18	1.81-8.54	4,25	1.16-7.34
70–79	3,78	3.30-4.25	1,56	1.15-1.98	3,46	-0.60-7.53	6,86	4.33-9.39	7,31	0.00-14.62	7,19	1.59-12.80	6,10	0.56-11.64
80–89	5,43	4.56-6.29	2,47	1.72-3.21	4,14	-1.41-9.69	16,50	7.63-25.36	10,5	-1.90-22.90	NA		8,76	-0.79-18.31
≥90	8,11	6.51-9.71	4,42	2.78-6.06	4,94	-2.52-12.40	NA		NA		NA		NA	
Sex, F	NS	NS	0,80	0.70 - 0.91	NS	NS	NS	NS	NS	NS	NS	NS	NS	NS
HM+HSCT	1,33	1.00 - 1.76	1,81	1.46 - 2.24	5,97	2.42 - 14.72	NS	NS	NS	NS	NS	NS	NS	NS
HM+neutropenia	1,58	1.33 - 1.87	1,36	1.14 - 1.62	5,86	2.64 - 13.01	NS	NS	NS	NS	8,49	2.32 - 31.06	NS	NS
HM	1,40	1.18 - 1.67	2,03	1.69 - 2.44	3,60	1.54 - 8.39	0,70	0.51 - 0.97	3,92	2.01 - 7.68	NS	NS	NS	NS
HIV/AIDS	NS	NS	NS	NS	NS	NS	NS	NS	NS	NS	NS	NS	NS	NS
Solid org.transplant	NS	NS	1,46	1.15 - 1.84	NS	NS	NS	NS	NS	NS	NS	NS	NS	NS
Solid Tumors	1,87	1.71 - 2.04	2,13	1.75 - 2.60	NS	NS	2,26	1.61 - 3.17	NS	NS	4,43	2.82 - 6.94	NS	NS
Diabetes	0,79	0.71 - 0.89	NS	NS	NS	NS	0,65	0.43 - 0.99	NS	NS	NS	NS	NS	NS
Respiratory diseases	NS	NS	0,74	0.61 - 0.91	NS	NS	NS	NS	NS	NS	NS	NS	NS	NS
Chronic renal failure	NS	NS	0,76	0.57 - 1.00	NS	NS	0,48	0.31 - 0.73	NS	NS	0,29	0.11 - 0.79	NS	NS
Cirrhosis	2,48	2.04 - 2.99	1,58	1.05 - 2.39	NS	NS	2,84	1.21 - 6.67	6,48	2.05 - 20.46	NS	NS	NS	NS
Acute renal failure	1,84	1.56 - 2.16	2,32	1.82 - 2.97	NS	NS	2,82	2.01 - 3.95	2,45	1.27 - 4.70	6,65	4.46 - 9.90	NS	NS
Surgery	0,60	0.54 - 0.66	NS	NS	NS	NS	NS	NS	NS	NS	NS	NS	NS	NS
Intensive care	NS	NS	4,55	3.86 - 5.36	12,69	6.82 - 23.63	6,91	5.19 - 9.20	8,49	4.57 - 15.74	8,73	6.49 - 11.76	12,50	7.64 - 20.45

HM, hematologic malignancy; HSCT, hematologic stem cell transplantation; NA, not applicable: no occurrences of infection in this risk group; NS, not statistically significant.
